# Supplementary material for: Distinct conformational states enable transglutaminase 2 to promote cancer cell survival versus cell death
Source: Commun Biol. 2024 Aug 13;7:982. doi: 10.1038/s42003-024-06672-x (PMC11319651; doi:10.1038/s42003-024-06672-x)
Supplement: Supplementary file 2 — Supplemental Material [file 42003_2024_6672_MOESM2_ESM.pdf]

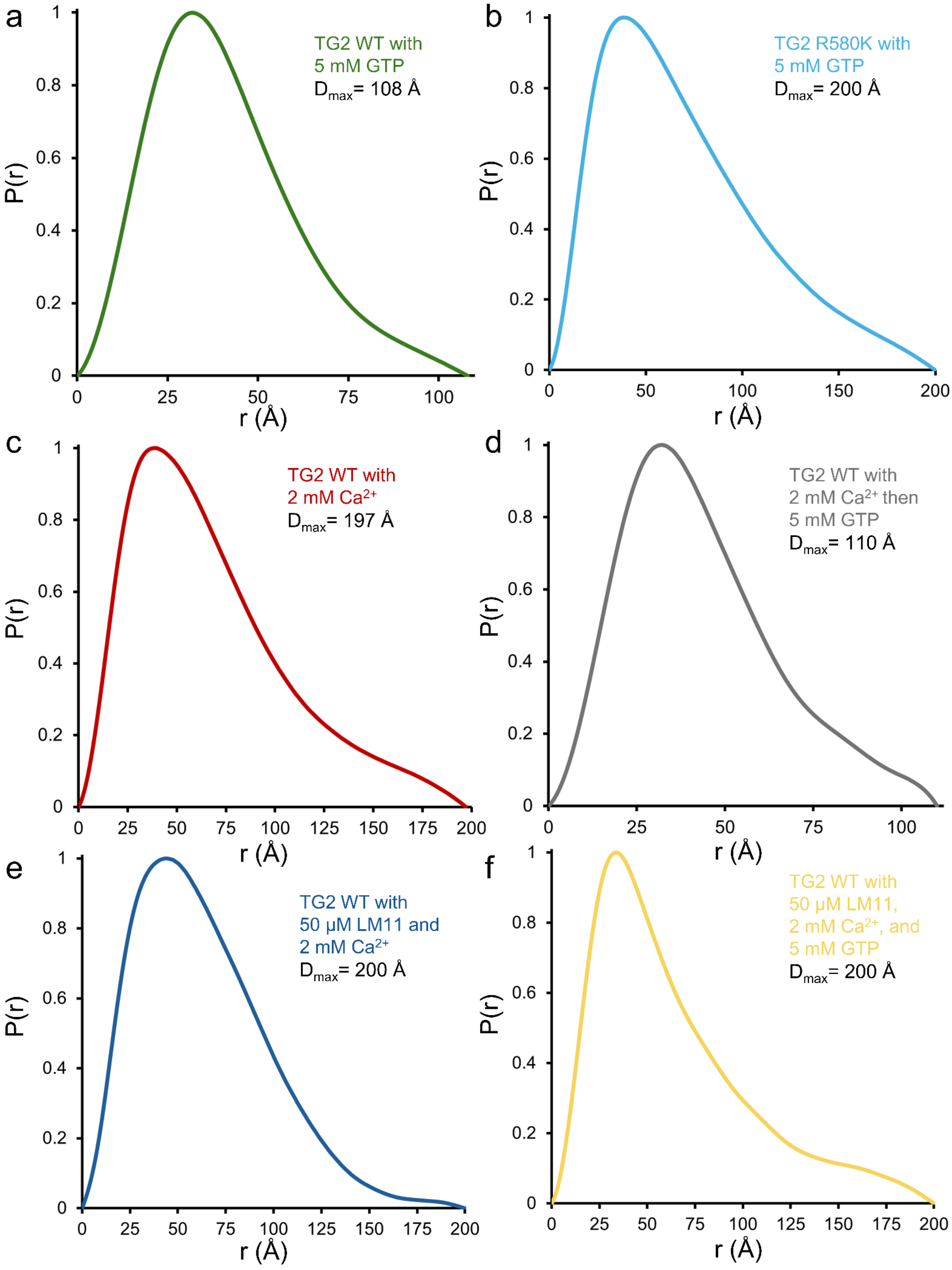

a
TG2 WT with
5 mM GTP
D_{max}= 108 Å
P(r)
r (Å)
b
TG2 R580K with
5 mM GTP
D_{max}= 200 Å
c
TG2 WT with
2 mM Ca^{2+}
D_{max}= 197 Å
d
TG2 WT with
2 mM Ca^{2+} then
5 mM GTP
D_{max}= 110 Å
e
TG2 WT with
50 μM LM11 and
2 mM Ca^{2+}
D_{max}= 200 Å
f
TG2 WT with
50 μM LM11,
2 mM Ca^{2+}, and
5 mM GTP
D_{max}= 200 Å

Figure S1: P(r) distributions and D_{max} for (a) TG2 WT with 5 mM GTP, (b) TG2 R580K with 5 mM GTP, (c) TG2 WT with 2 mM Ca^{2+}, (d) TG2 WT with 2 mM Ca^{2+}, then 5 mM GTP, (e) TG2 WT with 50 µM and LM11 2 mM Ca^{2+}, and (f) TG2 WT with 50 µM, 2 mM Ca^{2+}, and 5 mM GTP. All P(r) analysis was done using GNOM.

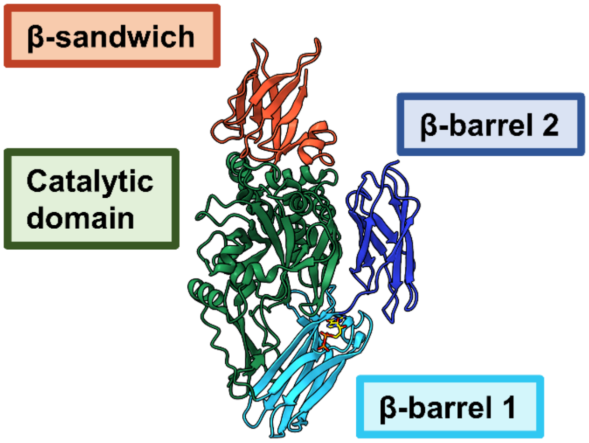

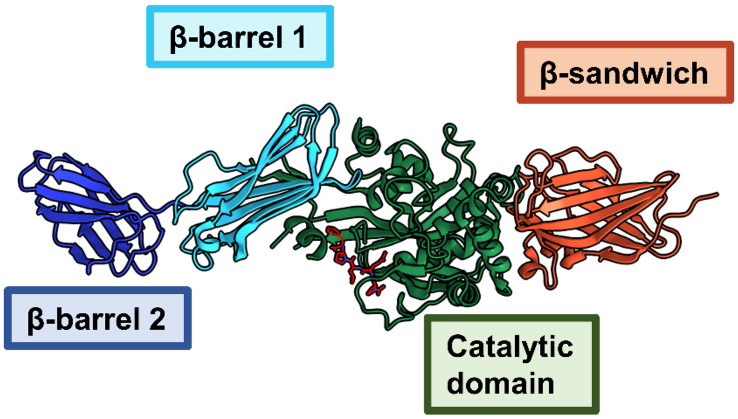

Closed state monomer

Cryo-EM, this study, PDB: 8TR9.
Calculated R_g (FoXS, Å): 29.2
Calculated D_{max} (FoXS, Å): 103
Residues in TG2 construct: 693 (Met1-Ala687 and N-terminal 6xHis tag)
Residues in Atomic Model: 686 (Glu3-Ala687)

Open state monomer

X-ray crystallography, PDB: 2Q3Z.
Calculated R_g (FoXS, Å): 38.4
Calculated D_{max} (FoXS, Å): 147
Residues in TG2 construct: 693 (Met1-Ala687 and N-terminal 6xHis tag)
Residues in Atomic Model: 655 (Met1-Asp306, Ser309-Asn318, Ser328-Gln406, Lys414-His461, Gly472-Ile683)

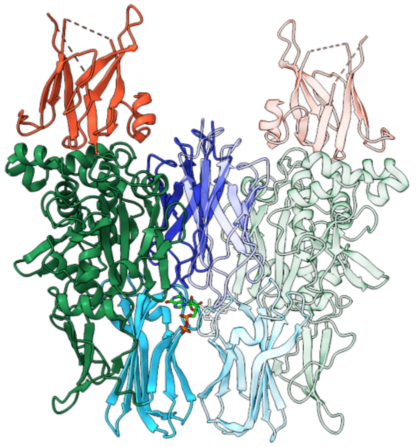

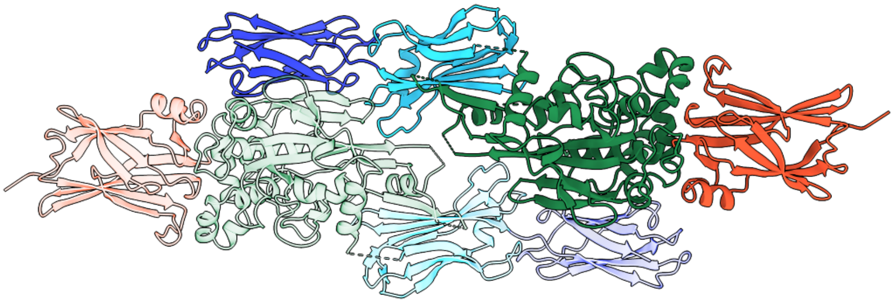

Closed state dimer

X-ray crystallography, PDB: 1KV3.
Calculated R_g (FoXS, Å): 35.6
Calculated D_{max} (FoXS, Å): 120
Residues in TG2 construct: 693 (Met1-Ala687 and N-terminal 6xHis tag)
Residues in Atomic Model: 1372 (2 x Glu3-Ala687)

Open state dimer

X-ray crystallography, PDB: 1KV3.
Calculated R_g (FoXS, Å): 46.4
Calculated D_{max} (FoXS, Å): 170
Residues in TG2 construct: 693 (Met1-Ala687 and N-terminal 6xHis tag)
Residues in Atomic Model: 1310 (2 x Met1-Asp306, Ser309-Asn318, Ser328-Gln406, Lys414-His461, Gly472-Ile683

Figure S2: Models used for analysis of TG2 using CRYSOL and OLIGOMER. The closed state monomer cryoEM structure (this study), the open state monomer crystal structure (PDB: 2Q3Z), the closed state dimer crystal structure (PDB: 1KV3) and the open state dimer crystal structure (PDB: 2Q3Z). Theoretical SAXS profiles of each atomic model was obtained using the FoXS Server, and the calculated R_g and D_{max} are included. In addition, the amino acid residue inventory is described for each atomic model.

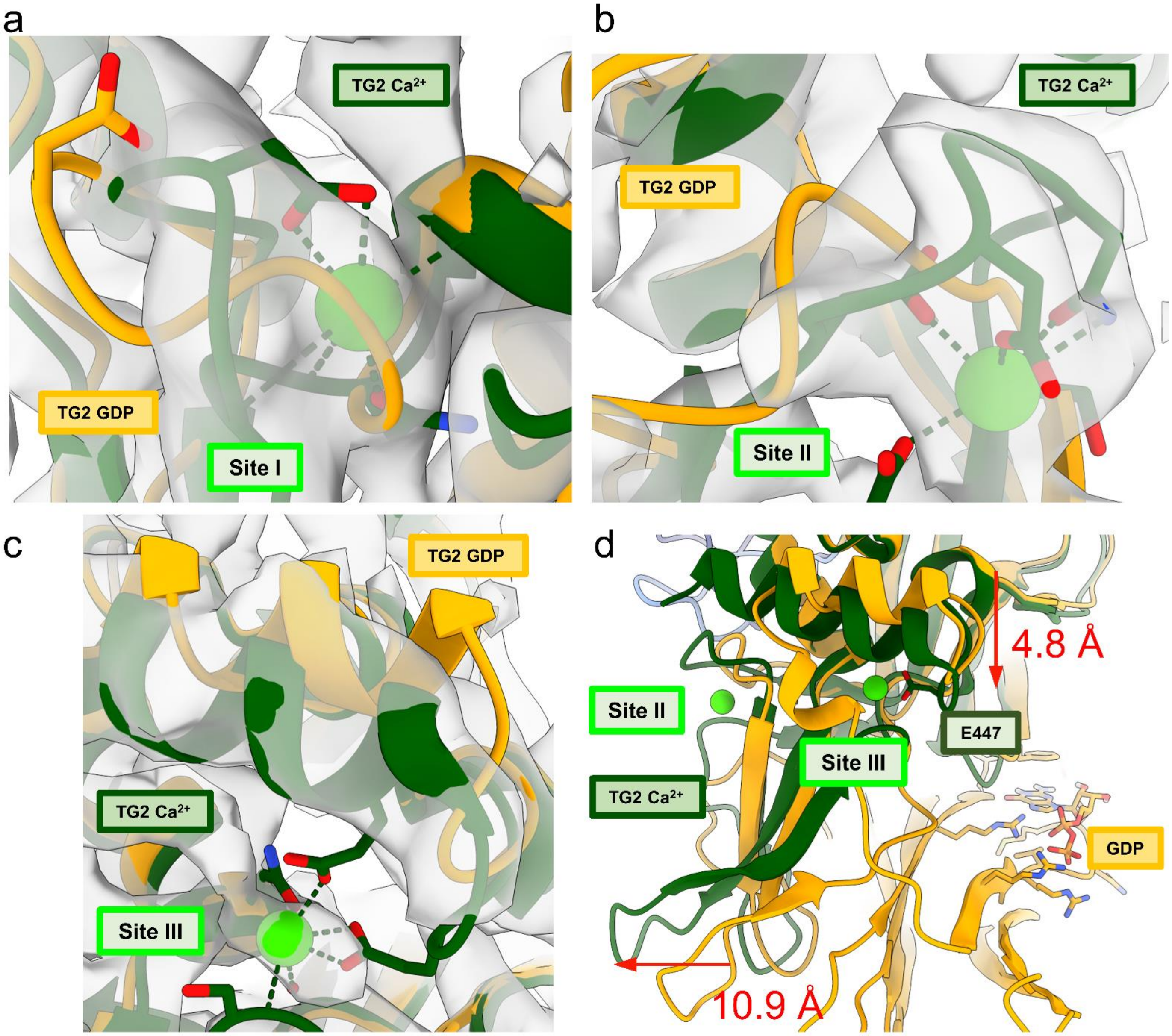
a
TG2 Ca2+
TG2 GDP
Site I
b
TG2 Ca2+
TG2 GDP
Site II
c
TG2 GDP
TG2 Ca2+
Site III
d
4.8 Å
Site II
E447
Site III
TG2 Ca2+
GDP
10.9 Å

Figure S3: (a-c) cryo-EM density compared to the atomic models of TG2 bound to GDP (orange) and TG2 bound to Ca^{2+} (green) for the Site I (a), Site II (b), and Site III (c) Ca^{2+} binding sites. (d) Conformational changes associated with Ca^{2+} binding to Site III increase GTP dissociation.

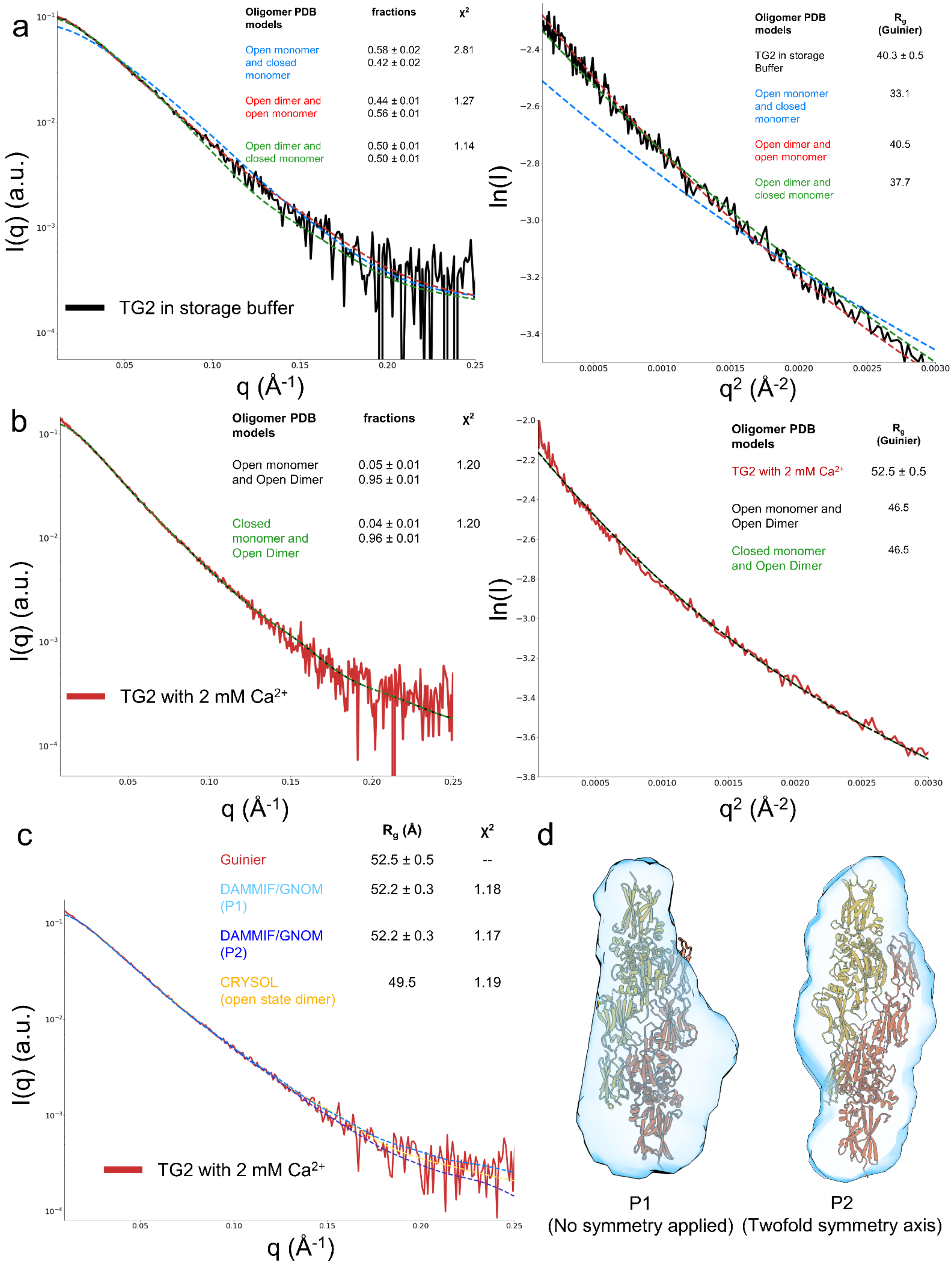

a
Oligomer PDB models
fractions
χ²
Open monomer and closed monomer
0.58 ± 0.02
0.42 ± 0.02
2.81
Open dimer and open monomer
0.44 ± 0.01
0.56 ± 0.01
1.27
Open dimer and closed monomer
0.50 ± 0.01
0.50 ± 0.01
1.14
TG2 in storage buffer
I(q) (a.u.)
q (Å⁻¹)
Oligomer PDB models
Rg (Guinier)
TG2 in storage Buffer
40.3 ± 0.5
Open monomer and closed monomer
33.1
Open dimer and open monomer
40.5
Open dimer and closed monomer
37.7
ln(I)
q² (Å⁻²)
b
Oligomer PDB models
fractions
χ²
Open monomer and Open Dimer
0.05 ± 0.01
0.95 ± 0.01
1.20
Closed monomer and Open Dimer
0.04 ± 0.01
0.96 ± 0.01
1.20
TG2 with 2 mM Ca2+
I(q) (a.u.)
q (Å⁻¹)
Oligomer PDB models
Rg (Guinier)
TG2 with 2 mM Ca2+
52.5 ± 0.5
Open monomer and Open Dimer
46.5
Closed monomer and Open Dimer
46.5
ln(I)
q² (Å⁻²)
c
Rg (Å)
χ²
Guinier
52.5 ± 0.5
--
DAMMIF/GNOM (P1)
52.2 ± 0.3
1.18
DAMMIF/GNOM (P2)
52.2 ± 0.3
1.17
CRYSOL (open state dimer)
49.5
1.19
TG2 with 2 mM Ca2+
I(q) (a.u.)
q (Å⁻¹)
d
P1
(No symmetry applied)
P2
(Twofold symmetry axis)

Figure S4: (a and b) Left: Analysis of TG2 in storage buffer (A) and with 2 mM Ca^{2+} (B) with OLIGOMER. The experimental scattering profiles were fit using combinations of the open state monomer crystal structure (PDB: 2Q3Z), the open state dimer model (this paper), and the closed state monomer cryoEM structure (this study). Right: The Guinier region of each plot with the Oligomer fits shown. (c) Quality of the DAMMIF analysis of TG2 with 2 mM Ca^{2+} assuming P1 (no symmetry, light blue) or P2 (twofold symmetry, dark blue). The CRYSOL fit of the open state dimer is included for comparison (yellow). (d) SAXS envelopes from DAMMIF bead models for P1 (no symmetry, left) and P2 (twofold symmetry, right). The open state dimer model is docked in for visualization.

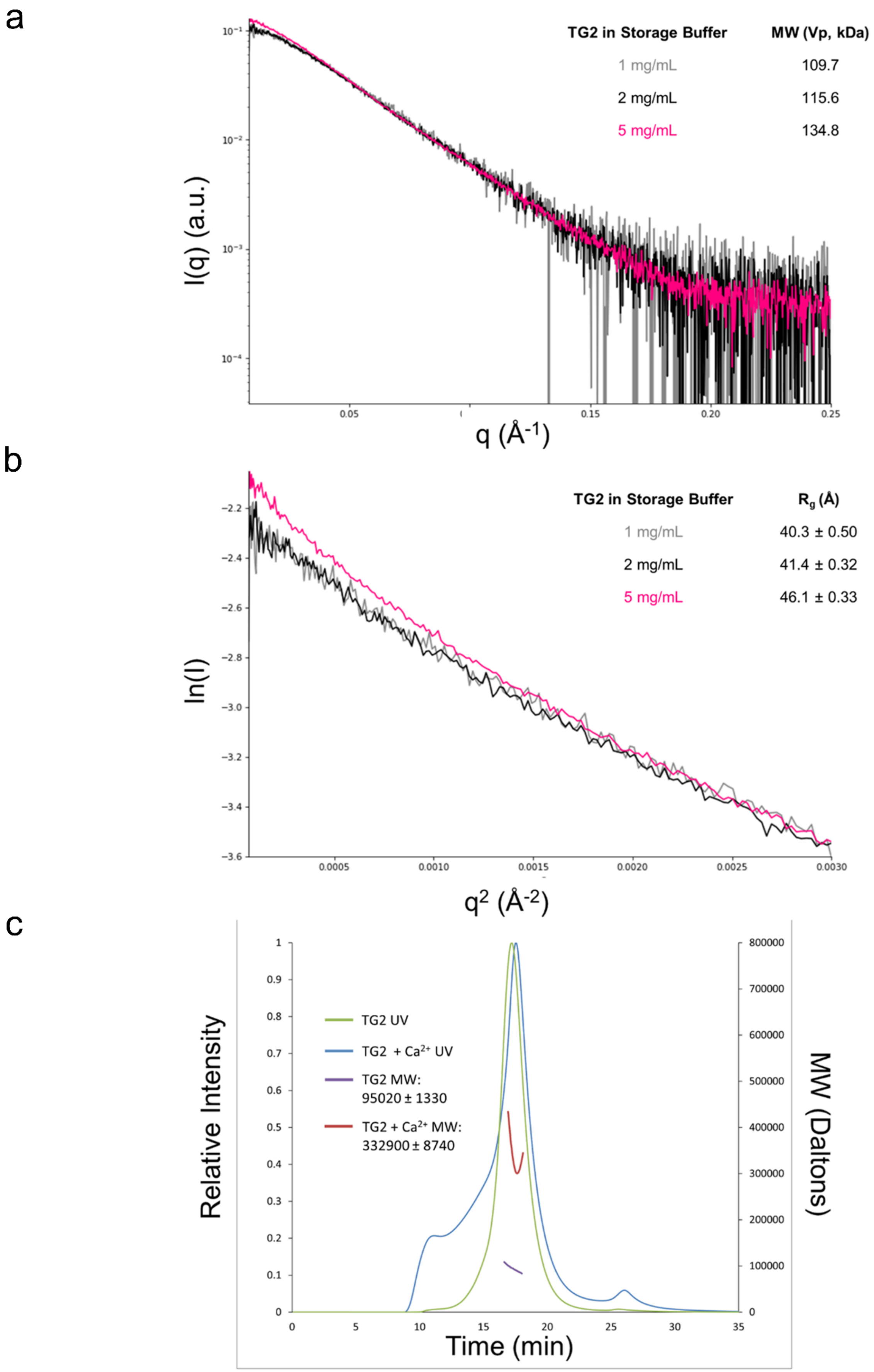

a
TG2 in Storage Buffer
MW (Vp, kDa)
1 mg/mL
109.7
2 mg/mL
115.6
5 mg/mL
134.8
I(q) (a.u.)
q (Å-1)
b
TG2 in Storage Buffer
Rg (Å)
1 mg/mL
40.3 ± 0.50
2 mg/mL
41.4 ± 0.32
5 mg/mL
46.1 ± 0.33
ln(I)
q2 (Å-2)
c
TG2 UV
TG2 + Ca2+ UV
TG2 MW:
95020 ± 1330
TG2 + Ca2+ MW:
332900 ± 8740
Relative Intensity
MW (Daltons)
Time (min)

Figure S5: (a) Scattering profiles of TG2 in storage buffer at different concentrations. (b) Guinier Plot of TG2 in storage buffer at different concentrations. (c) SEC-MALS measurements of TG2 in storage buffer and in 2 mM Ca^{2+}

LM1

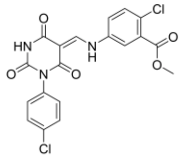

LM4

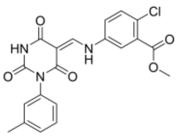

LM7

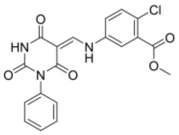

LM10

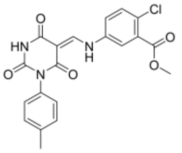

LM2

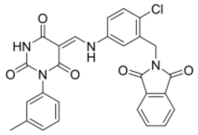

LM5

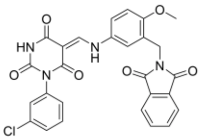

LM8

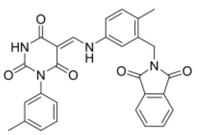

LM11

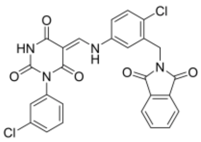

LM3

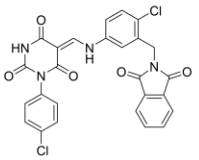

LM6

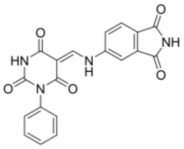

LM9

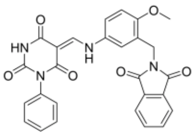

LM12

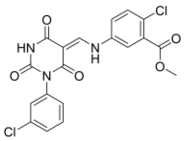

Figure S6: Chemical structures of the LM series of inhibitors.

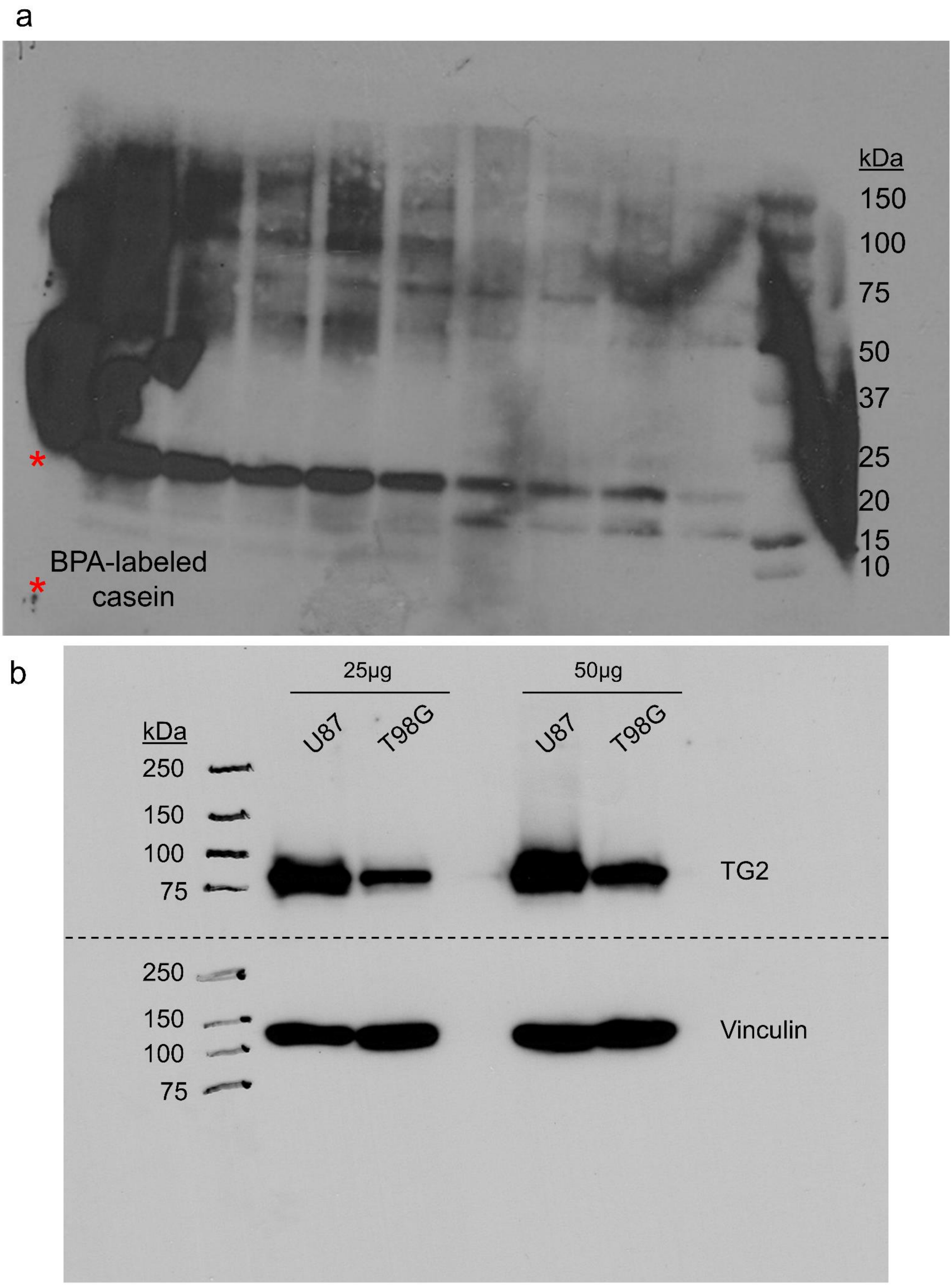
a
kDa
150
100
75
50
37
25
20
15
10
*
* BPA-labeled casein
b
25µg
50µg
U87
T98G
U87
T98G
kDa
250
150
100
75
TG2
250
150
100
75
Vinculin

Figure S7: Full gel images from the manuscript. (a) Gel image of the inhibition of TG2 crosslinking activity shown in Fig. 5a. (b) Gel image of the western blot for TG2 expression in U87 and T98G cells shown in Fig. 5c.

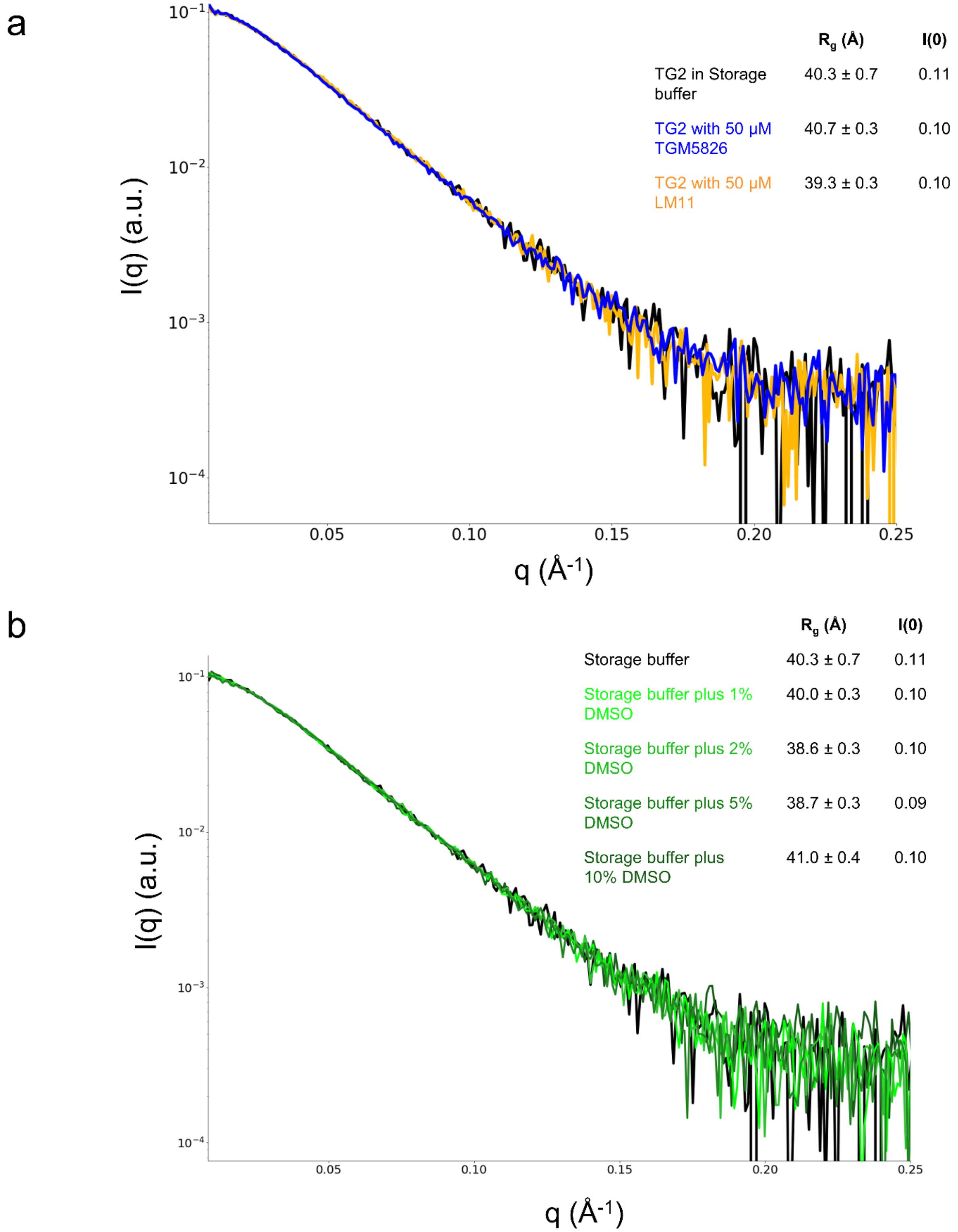
a
Rg (Å)
I(0)
TG2 in Storage buffer
40.3 ± 0.7
0.11
TG2 with 50 μM TGM5826
40.7 ± 0.3
0.10
TG2 with 50 μM LM11
39.3 ± 0.3
0.10
I(q) (a.u.)
q (Å-1)
b
Rg (Å)
I(0)
Storage buffer
40.3 ± 0.7
0.11
Storage buffer plus 1% DMSO
40.0 ± 0.3
0.10
Storage buffer plus 2% DMSO
38.6 ± 0.3
0.10
Storage buffer plus 5% DMSO
38.7 ± 0.3
0.09
Storage buffer plus 10% DMSO
41.0 ± 0.4
0.10
I(q) (a.u.)
q (Å-1)

Figure S8: (a) Scattering profiles of TG2 treated with LM11 and TTGM 5826. The profiles of the LM11 bound (orange) and TTGM 5826 bound (blue) can be compared to TG2 in storage buffer (black) to show that no conformational change is associated with drug binding in the absence of calcium. (b) Scattering profiles of TG2 with different amounts of added DMSO. Both TTG< 5826 and LM11 are stored in DMSO so these control experiments help show that DMSO is not causing any TG2 conformational changes.

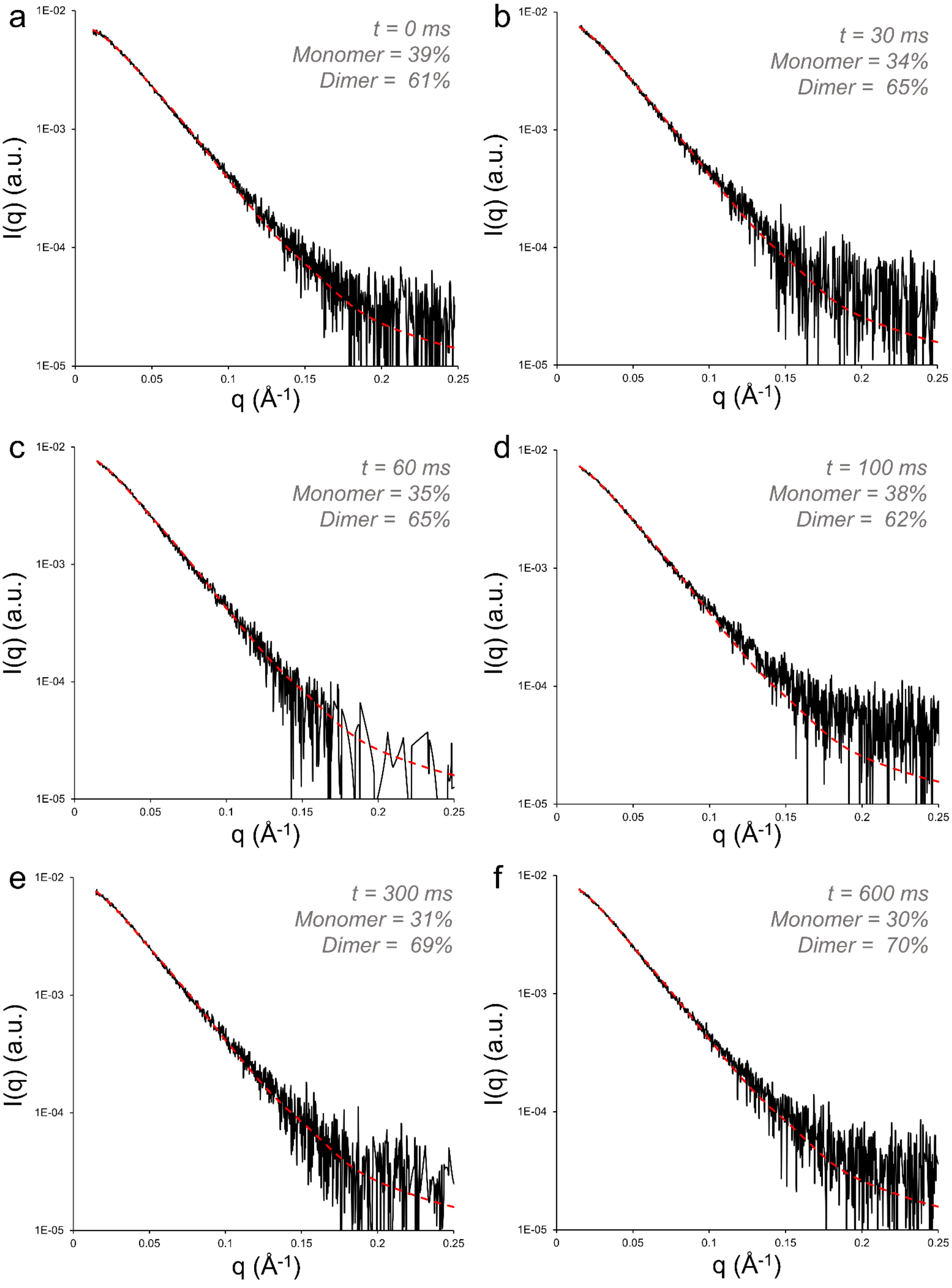

a
t = 0 ms
Monomer = 39%
Dimer = 61%
b
t = 30 ms
Monomer = 34%
Dimer = 65%
c
t = 60 ms
Monomer = 35%
Dimer = 65%
d
t = 100 ms
Monomer = 38%
Dimer = 62%
e
t = 300 ms
Monomer = 31%
Dimer = 69%
f
t = 600 ms
Monomer = 30%
Dimer = 70%
I(q) (a.u.)
q (Å-1)
1E-02
1E-03
1E-04
1E-05
0
0.05
0.1
0.15
0.2
0.25

Figure S9: SAXS deconvolution fitting of the time-resolved scattering of TG2 mixed with calcium. The closed state monomer and open state dimer were used as inputs and the quality of the fit was evaluated with the standard error of the fit. For each timepoint, the scattering profile is shown in black, and the fit is shown in red.

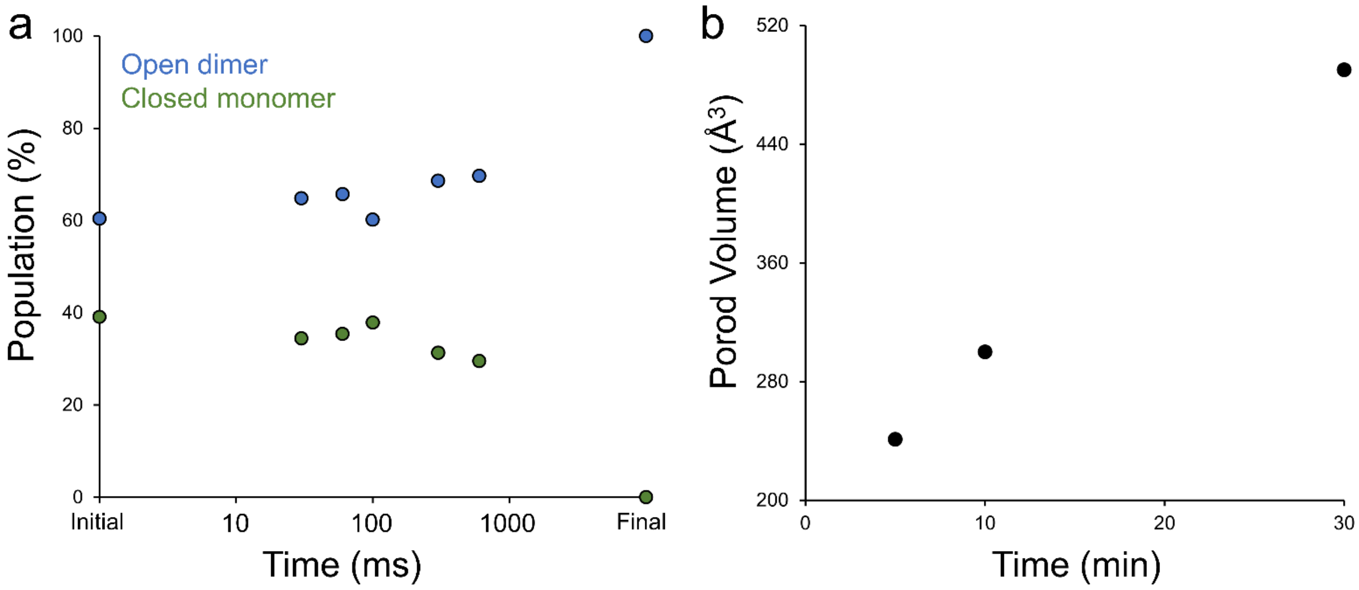

a
b
Open dimer
Closed monomer
Population (%)
0
20
40
60
80
100
Initial
10
100
1000
Final
Time (ms)
Porod Volume (Å^3)
200
280
360
440
520
0
10
20
30
Time (min)

Figure S10: (a) The relative populations of open dimer (blue) and closed monomer (green) in the presence of Ca^{2+} as a function of time. (b) The Porod volume of TG2 treated with Ca^{2+} continues to increase on long time scales (minutes).

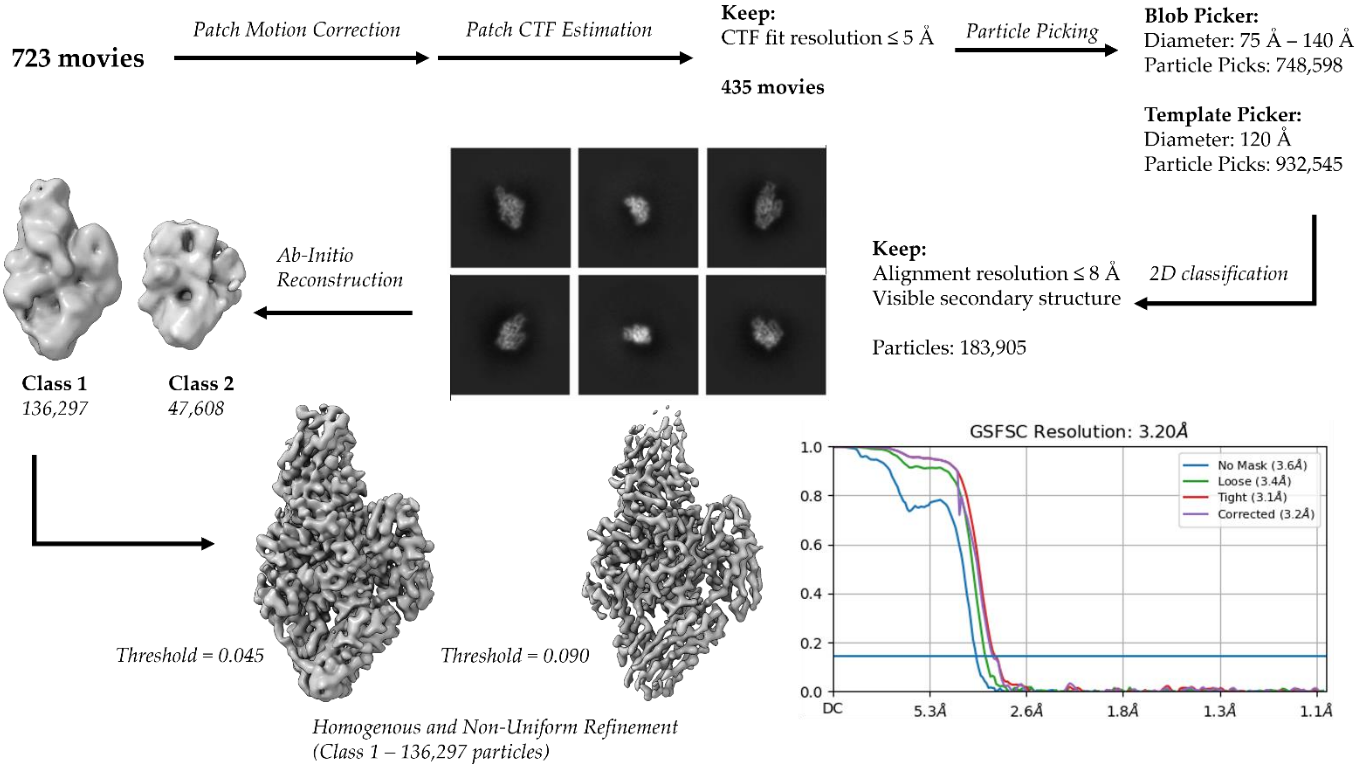
723 movies
Patch Motion Correction
Patch CTF Estimation
Keep:
CTF fit resolution ≤ 5 Å
435 movies
Particle Picking
Blob Picker:
Diameter: 75 Å – 140 Å
Particle Picks: 748,598
Template Picker:
Diameter: 120 Å
Particle Picks: 932,545
2D classification
Keep:
Alignment resolution ≤ 8 Å
Visible secondary structure
Particles: 183,905
Ab-Initio
Reconstruction
Class 1
136,297
Class 2
47,608
Threshold = 0.045
Threshold = 0.090
Homogenous and Non-Uniform Refinement
(Class 1 – 136,297 particles)
GSFSC Resolution: 3.20Å
No Mask (3.6Å)
Loose (3.4Å)
Tight (3.1Å)
Corrected (3.2Å)
1.0
0.8
0.6
0.4
0.2
0.0
DC
5.3Å
2.6Å
1.8Å
1.3Å
1.1Å

Figure S11: Cryo-EM data processing workflow for TG2 bound to GDP.

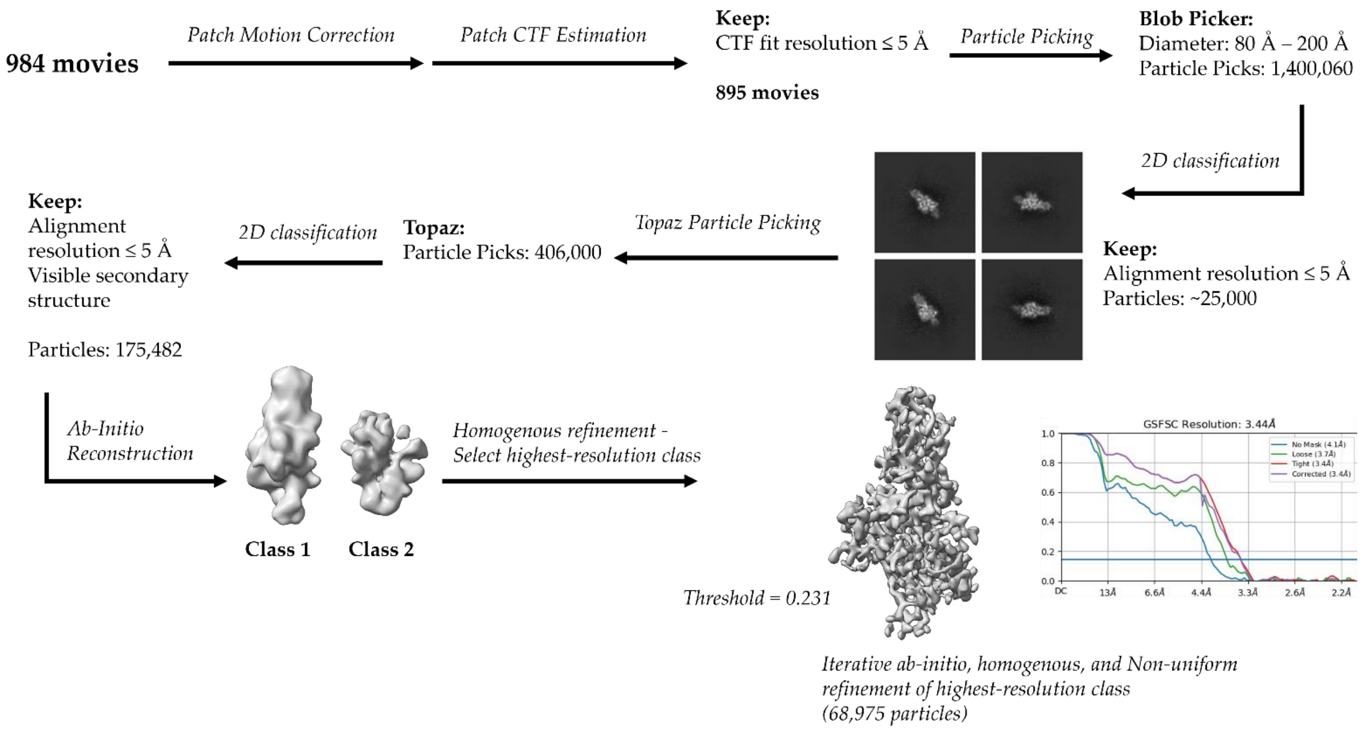
984 movies
Patch Motion Correction
Patch CTF Estimation
Keep:
CTF fit resolution ≤ 5 Å
895 movies
Particle Picking
Blob Picker:
Diameter: 80 Å – 200 Å
Particle Picks: 1,400,060
2D classification
Keep:
Alignment resolution ≤ 5 Å
Particles: ~25,000
Topaz Particle Picking
Topaz:
Particle Picks: 406,000
2D classification
Keep:
Alignment resolution ≤ 5 Å
Visible secondary structure
Particles: 175,482
Ab-Initio Reconstruction
Class 1
Class 2
Homogenous refinement - Select highest-resolution class
Threshold = 0.231
GSFSC Resolution: 3.44Å
No Mask (4.1Å)
Loose (3.7Å)
Tight (3.4Å)
Corrected (3.4Å)
Iterative ab-initio, homogenous, and Non-uniform refinement of highest-resolution class
(68,975 particles)

Figure S12: Cryo-EM data processing workflow for TG2 bound to calcium.

Table S1: Summary of Guinier and GNOM fits of static SAXS profiles.

**SAXS Profile**	**Rg - Guinier (Å)**	**I(0) - Guinier (arbitrary units)**	**Rg - GNOM (Å)**	**D_{max} (Å)**	**Ambimeter**
2 mg/mL TG2	40.3 ± 0.3	0.11 ± 0.001	42.5 ± 0.3	148	1.9
TG2 + 5 mM GTP	31.9 ± 0.2	0.09 ± 0.0005	32.4 ± 0.2	108	1.0
TG2 + 2 mM Ca^{2+}	52.5 ± 0.5	0.14 ± 0.001	54.6 ± 0.4	197	0.8
TG2 + 2 mM Ca^{2+} and 5 mM GTP	34.2 ± 0.3	0.09 ± 0.0004	34.1 ± 0.2	110	0.9
TG2 + 50 μM LM11	39.3 ± 0.3	0.09 ± 0.0005	40.6 ± 0.2	140	1.9
TG2 + 2 mM Ca^{2+} and 50 μM LM11	60.2 ± 1.1	0.18 ± 0.003	49.9 ± 0.6	200	2.4
TG2 + 2 mM Ca^{2+}, 50 μM LM11, and 5 mM GTP	61.6 ± 1.1	0.16 ± 0.002	59.5 ± 0.5	200	1.5

Table S2: Predicted TG2 calcium binding sites[1].

**Site**	**Reason for prediction**	**Interacting residues**	**Confirmed by cryo-EM structure (Y/N)**
S1	Homology with TG3	228 V**N**C**NDD**QGV	Y
S2A	Homology with FXIIIa and TG3	395 A**E**V**N**A**D**V	Y
S2B	Homology with FXIIIa and TG3	445 YP**E**GSS**EE**R**E**A	
S3A	Homology with TG3	305 H**D**Q**N**S**N**L	Y
S3B	Homology with TG3	326 DKS**E**MIWN	
S4	Highly negative surface charge	149 YL**D**S**EEE**RQ**E**Y	N
S5	Highly negative surface charge	432 GR**DE**R**ED**IT	N

Table S3: Crosslinking IC50 and docking analysis of LM series in comparison to TTGM 5826.

Molecule	Crosslinking IC50 (µM)	Caco2 Permeability	MDCK Permeability
LM1	n.d.	122.5	126.2
LM2	141	92.0	68.9
LM3	60	125.6	276.6
LM4	> 300	153.1	138.4
LM5	305	88.6	88.9
LM6	> 300	25.8	9.5
LM7	> 300	150.9	136.3
LM8	> 300	66.6	26.4
LM9	> 300	106.0	43.7
LM10	> 300	134.3	116.3
LM11	10	89.8	164.0
LM12	> 300	141.3	302.6
5826	34	67.3	26.8

Table S4: Calcium series TR-SAXS analysis by Guinier and molecular weight estimate from Shape and Size. The theoretical molecular weight for a TG2 monomer and dimer are included in the first two rows for comparison.

Timepoint (ms)	Rg (Å)	I(0)	Molecular Weight (S&S - kDa)
NA	NA	NA	Monomer = 77 kDa
NA	NA	NA	Dimer = 154 kDa
0	40.6 ± 0.4	9e-6	108
32	39.9 ± 0.8	9e-6	107
63	41.3 ± 0.5	9e-6	98
100	40.7 ± 0.4	9e-6	103
316	45.1 ± 0.6	10e-6	122
631	45.8 ± 0.5	10e-6	129

Table S5: LM11 and calcium series TR-SAXS analysis by Guinier and molecular weight estimate from Shape and Size. The theoretical molecular weight for a TG2 monomer and dimer are included in the first two rows for comparison.

Timepoint (ms)	Rg (Å)	I(0)	Molecular Weight (S&S - kDa)
NA	NA	NA	Monomer = 77 kDa
NA	NA	NA	Dimer = 154 kDa
0	40.6 ± 0.4	9e-6	108
100	45.2 ± 0.7	10e-6	110
316	46.8 ± 0.7	10e-6	118
631	50.9 ± 0.5	10e-6	135

Table S6: SASBDB accession codes and description

SASDTL3	Tissue Transglutaminase: Equilibrium
SASDTM3	Tissue Transglutaminase + 25 µM GTP
SASDTN3	Tissue Transglutaminase + 50 µM GTP
SASDTP3	Tissue Transglutaminase + 100 µM GTP
SASDTQ3	Tissue Transglutaminase + 250 µM GTP
SASDTR3	Tissue Transglutaminase + 500 µM GTP
SASDTS3	Tissue Transglutaminase + 1 mM GTP
SASDTT3	Tissue Transglutaminase + 2 mM GTP
SASDTU3	Tissue Transglutaminase + 5 mM GTP: Equilibrium
SASDTV3	Tissue Transglutaminase + 5 mM GDP
SASDTW3	Tissue Transglutaminase + 250 µM CaCl2
SASDTX3	Tissue Transglutaminase + 500 µM CaCl2
SASDTY3	Tissue Transglutaminase + 1 mM CaCl2
SASDTZ3	Tissue Transglutaminase + 2 mM CaCl2
SASDT24	Tissue Transglutaminase + 50 µM inhibitor LM11
SASDT34	Tissue Transglutaminase + 50 µM inhibitor LM11 + 250 µM CaCl2
SASDT44	Tissue Transglutaminase + 50 µM inhibitor LM11 + 500 µM CaCl2
SASDT54	Tissue Transglutaminase + 50 µM inhibitor LM11 + 1 mM CaCl2
SASDT64	Tissue Transglutaminase + 50 µM inhibitor LM11 + 2 mM CaCl2
SASDT74	Tissue Transglutaminase + CaCl2 + inhibitor LM11: Equilibrium
SASDT84	Tissue Transglutaminase + inhibitor LM11 + CaCl2: Time-resolved 100 ms
SASDT94	Tissue Transglutaminase + inhibitor LM11 + CaCl2: Time-resolved 316 ms
SASDTA4	Tissue Transglutaminase + inhibitor LM11 + CaCl2: Time-resolved 631 ms
SASDTB4	Tissue Transglutaminase + CaCl2 + inhibitor LM11 + GTP: Equilibrium
SASDTC4	Tissue Transglutaminase + 50 µM inhibitor 5826 (1% DMSO)
SASDTD4	Tissue Transglutaminase + 50 µM inhibitor 5826 (2% DMSO)
SASDTE4	Tissue Transglutaminase + 100 µM inhibitor 5826 (2% DMSO)
SASDTF4	Tissue Transglutaminase + CaCl2 + inhibitor 5826: Equilibrium
SASDTG4	Tissue Transglutaminase + CaCl2 + inhibitor 5826 + GTP: Equilibrium
SASDTH4	Tissue Transglutaminase + 1% DMSO
SASDTJ4	Tissue Transglutaminase + 2% DMSO
SASDTK4	Tissue Transglutaminase + 5% DMSO
SASDTL4	Tissue Transglutaminase + 10% DMSO
SASDTM4	Tissue Transglutaminase R580K mutant
SASDTN4	Tissue Transglutaminase R580K mutant + 100 µM GTP
SASDTP4	Tissue Transglutaminase R580K mutant + 1 mM GTP
SASDTQ4	Tissue Transglutaminase R580K mutant + 2 mM GTP
SASDTR4	Tissue Transglutaminase R580K mutant + 5 mM GTP

TR-SAXS

Script to define function

```
function err = TG_unmix(params,q,I,drawplot)

a = params(1)
b = params(2)

I_1 = interp1(monomer_open(:,1),monomer_open(:,2),q,'linear');
I_2 = interp1(dimer_open(:,1),dimer_open(:,2),q,'linear');

I_a = I_1/sum(I_1);
I_b = I_2/sum(I_2);
I_data = I/sum(I);

I_data_f = a*I_a + b*I_b;

funcerr = I_data_f - I_data;

err = sum(funcerr.^2);

% Plotting

qmin = min(q); qmax = max(q);

if exist('drawplot') == 1
    subplot(221);
    semilogy(q,I_data,'o',q,I_data_f);
    legend('Data','Fit'); %axis tight;
    title(['a = ' num2str(params(1)) ', b = '  num2str(params(2)) ]);
    xlabel('q (1/A)');

    subplot(222);
    plot(q,I_data,q,a*I_a,q,b*I_b); %axis tight
    %title([date]);
    title('Components')
    xlabel('q (1/A)');
    legend('Data','open monomer','open dimer');

end
```

Script to use function – applied to the 300 ms timepoint with calcium as an example

```
params = fminsearch('TG_unmix_',[0.5 0.5],[],tg_ca_300ms(15:1007,1),
tg_ca_300ms(15:1007,2),tg_ca_300ms(15:1007,3));
figure; TG_unmix(params,tg_ca_300ms(15:807,1),tg_ca_300ms(15:807,2),1);
```

References

1 Király, R. *et al.* Functional significance of five noncanonical Ca2+-binding sites of human transglutaminase 2 characterized by site-directed mutagenesis. *FEBS Journal* **276**, 7083-7096, doi:10.1111/j.1742-4658.2009.07420.x (2009).
